# Conservative Management of Cesarean Scar Ectopic Pregnancy with Fetal Heart Activity and a Very High *β*-hCG

**DOI:** 10.1155/2015/959876

**Published:** 2015-12-06

**Authors:** Rodney McLaren, Sandra McCalla, Mohamad Irani

**Affiliations:** ^1^Department of Obstetrics and Gynecology, Maimonides Medical Center, Brooklyn, NY 11219, USA; ^2^Ronald O. Perelman and Claudia Cohen Center for Reproductive Medicine, Weill Cornell Medical Center, New York, NY 10021, USA

## Abstract

Cesarean scar ectopic pregnancy (CSP) is a rare type of ectopic pregnancy that is growing in incidence. The diagnosis of most CSP occurs when patients present in unstable conditions requiring surgical management and leading sometimes to hysterectomy. It has been shown that medical management is a safe option for early diagnosed hemodynamically stable CSP. However, no cases of CSP with *β*-hCG higher than 62,000 IU/L, conservatively treated, have been reported. We report the case of a 29-year-old patient who presented for her first prenatal visit at 13-week gestation and was diagnosed with CSP with present fetal heart tones and a quantitative *β*-hCG of 144,337 IU/L. She was treated with bilateral uterine artery embolization and systemic methotrexate. Her *β*-hCG significantly decreased and became undetectable within 10 weeks. We propose that patients with CSP with very high *β*-hCG and fetal heart activity can be offered conservative or fertility preserving management.

## 1. Introduction

Cesarean scar ectopic pregnancy (CSP) is considered one of the rarest types of ectopic pregnancies. Its incidence has been reported as 1/2200–1/1800 pregnancies [1]. However, this incidence is increasing possibly due to the increase in cesarean section rates. Early diagnosis is prudent to avoid severe complications such as uterine rupture and severe hemorrhage. The diagnosis is usually made via ultrasound, showing the following criteria: (1) an empty uterine cavity and cervical canal, (2) a gestational sac located anteriorly at the isthmus, and (3) evidence of a functional trophoblastic/placental circulation on color Doppler [2].

To date there are no guidelines on the optimal treatment of CSP in patients who are hemodynamically stable. There are many conservative treatment modalities described in the literature including systematic methotrexate, local methotrexate, bilateral uterine arteries embolization (UAE), and combined UAE and local methotrexate. However, there are no previous reports of conservative management for advanced CSP with extremely high *β*-hCG level (>100,000 IU/L) and detected fetal heart activity. To our knowledge, we are reporting the first case of CSP with *β*-hCG of 144,337 IU/L and a detected fetal heart activity that was successfully treated with uterine artery embolization and systemic methotrexate.

## 2. Case Report

**Figure 1:**
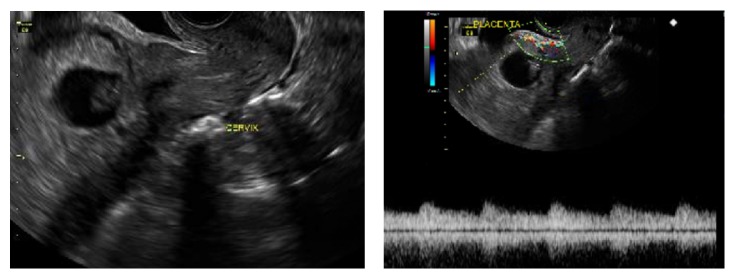
Transvaginal ultrasound showing cesarean scar ectopic pregnancy with placental circulation seen on color Doppler.

**Figure 2:**
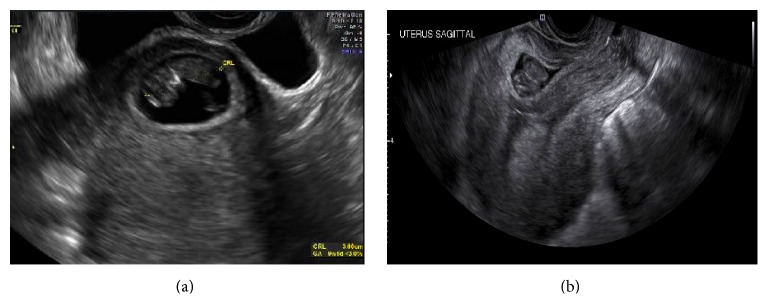
Transvaginal ultrasound showing fetus before treatment (a) and 7 days following uterine artery embolization and systemic methotrexate (b).

A 29-year-old woman G5P2103 (2 term deliveries, 1 preterm delivery, 0 miscarriage, and 3 living children) presented at 13-week gestation for her first prenatal visit. Patient had no complaints at the time. Patient has a history of two prior cesarean sections after a term normal vaginal delivery. The primary cesarean section was at 31 weeks for placenta previa and last cesarean section was at term in 2012. Patient has no medical history other than the aforementioned. She was dated by her last menstrual period (LMP). She reported regular menses, though she was not definite about her LMP. During this visit, pelvic ultrasound was performed showing a cesarean scar ectopic pregnancy measuring 9 weeks and 6 days by crown-rump length with detected fetal heart activity and fetal movement (Figures 1 and 2). The urinary bladder wall appeared intact. On examination, patient's vital signs were within normal limits. Her abdomen was soft, nontender, and not distended. There was no vaginal bleeding and the cervix was closed on speculum examination. Patient was then sent to the hospital for admission due to risk of uterine rupture and severe hemorrhage.

Initial laboratory tests revealed a *β*-hCG of 144,337 IU/L. The hemoglobin, white blood cell count, platelets, creatinine, and liver function enzymes were within normal limits. She was Rhesus positive.

Patient was counseled about the prognosis of CSP and the treatment options. Patient desired to preserve her fertility and opted for conservative management. The decision was made to treat her with a systemic single dose regimen of methotrexate 85 mg IM (50 mg/m^2^) along with uterine artery embolization (UAE). The endovascular embolization of the uterine arteries via the right common femoral artery was successfully performed by an interventional radiologist using Gelfoam slurry.

On day 4 after embolization and first dose of methotrexate, the *β*-hCG was 41,843 IU/L, which was significantly lower than baseline. It was decided that it was safe for the patient to be discharged home and followed up on day 7 for a repeat *β*-hCG and sonogram.

On day 7, patient continued to be asymptomatic. The repeat ultrasound revealed an amorphous structure of low echogenicity in place of the gestational sac and fetus (Figure 2). *β*-hCG was 12,379 IU/L.

**Figure 3:**
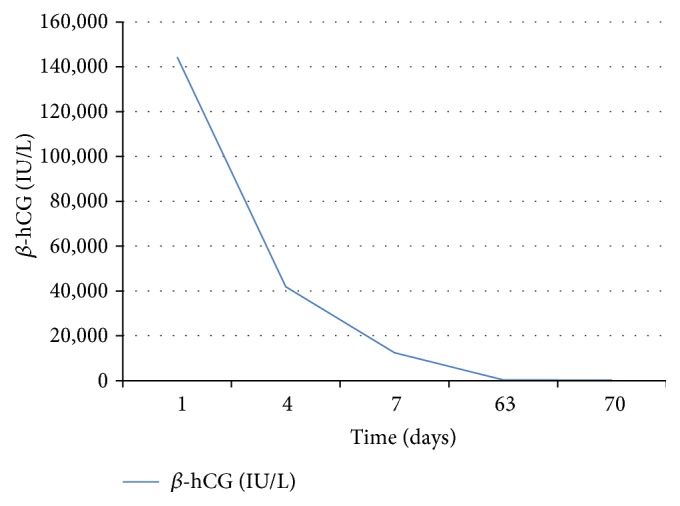
Graph depicting the trend of serum *β*-hCG level following uterine artery embolization and systemic methotrexate.

Patient continued follow-up weekly in the office until the *β*-hCG was undetectable within 10 weeks (Figure 3).

## 3. Discussion

Cesarean scar ectopic pregnancy is a rare type of ectopic pregnancy. Prompt diagnosis and treatment are essential to preserve the uterus and reduce the risk of uterine rupture and severe hemorrhage. Most cases of undiagnosed cesarean scar ectopic pregnancies necessitate hysterectomy due to late presentation of unstable patients. The most common presenting symptom is painless vaginal bleeding (38%) followed closely by not having a presenting symptom (36%). Other less common presenting symptoms are abdominal pain with bleeding (16%) and solely abdominal pain (9%). The diagnosis is made by transvaginal ultrasound with a documented 85% sensitivity [1]. Some authors report the use of magnetic resonance imaging if transvaginal ultrasound fails to identify typical findings of CSP [3].

The reported operative treatments for CSP are dilation and curettage (D&C) and laparotomy or laparoscopy to excise trophoblastic tissues [4]. However, there are multiple types of conservative treatments, which include systemic and local methotrexate, uterine artery embolization, use of local embryocides such as potassium chloride, and sac aspiration as well as combinations thereof [1, 5].

Systemic methotrexate alone for CSP has a high failure rate reaching 57% of cases [6]. A retrospective cohort study including 119 cases of CSP compared systemic methotrexate followed by dilation and curettage with uterine artery embolization followed by D&C [7]. Systemic methotrexate followed by D&C resulted in slower declines of *β*-hCG levels and greater blood loss at curettage and required additional interventions.

UAE with local methotrexate has been proven to be effective in the treatment of cesarean scar ectopic pregnancy and has been suggested as an initial treatment option [6]. In women who undergo UAE, local administration of methotrexate has been suggested to be a better route compared to its systemic administration because of the theoretical reduced efficacy of methotrexate resulting from the decrease blood supply to the pregnancy. Yang et al. also demonstrated the increased effectiveness and safety of uterine artery embolization with local methotrexate versus other treatment modalities [8].

Krissi et al. demonstrated the effectiveness of uterine artery methotrexate followed by embolization and systemic methotrexate in nontubal ectopic pregnancies [9]. Of the 6 CSP, the highest *β*-hCG in their study was 28,484 IU/L. Yang et al. also demonstrated the increased effectiveness and safety of UAE with local methotrexate versus other treatment modalities even with initial *β*-hCG >50,000 IU/L [8]. The highest *β*-hCG conservatively treated successfully was 62,000 IU/L [10].

To date these treatment modalities have been effective in patients with *β*-hCG no higher than 62,000 IU/L [10]. Our case describes conservative treatment with systemic methotrexate plus uterine artery embolization in a CSP with *β*-hCG of >140,000 IU/L. It demonstrates the success of conservative management when initial *β*-hCG level is markedly elevated. Patients should receive precautions and be counseled about the possibility of failure and the need for emergency surgery should massive hemorrhage ensue.

In summary, this case illustrates that *β*-hCG level greater than 100,000 IU/L does not preclude conservative (nonsurgical fertility preserving) management of cesarean scar ectopic pregnancy. Close monitoring of response to treatment and its complications and thorough counseling about alternatives are essential requisites for such management.
